# Investigating the Anti-inflammatory Effect of Quinoline Derivative: N1-(5-methyl-5H-indolo[2,3-b]quinolin-11-yl)benzene-1,4-diamine Hydrochloride Loaded Soluble Starch Nanoparticles Against Methotrexate-induced Inflammation in Experimental Model

**DOI:** 10.1186/s12575-024-00240-7

**Published:** 2024-06-03

**Authors:** Dalia Medhat, Mona A. El-Bana, Ibrahim El-Tantawy El-Sayed, Abdullah A. S. Ahmed, Mehrez E. El-Naggar, Jihan Hussein

**Affiliations:** 1 Medical Biochemistry Department, Medical Research and Clinical Studies Institute, National Research Centre, 12622 Dokki, Giza, Egypt; 2 Chemistry Department, Faculty of Science, Menoufia University, 32511 Shebin El Koom, Egypt; 3 Institute of Textile Research and Technology, National Research Centre, 12622 Dokki, Giza, Egypt

**Keywords:** Quinoline derivative, Soluble starch nanoparticles, Methotrexate, Homocysteine

## Abstract

**Background:**

It is necessary to develop advanced therapies utilizing natural ingredients with anti-inflammatory qualities in order to lessen the negative effects of chemotherapeutics.

**Results:**

The bioactive N1-(5-methyl-5H-indolo[2,3-b]quinolin-11-yl)benzene-1,4-diamine hydrochloride (NIQBD) was synthesized. After that, soluble starch nanoparticles (StNPs) was used as a carrier for the synthesized NIQBD with different concentrations (50 mg, 100 mg, and 200 mg). The obtained StNPs loaded with different concentrations of NIQBD were coded as StNPs-1, StNPs-2, and StNPs-3. It was observed that, StNPs-1, StNPs-2, and StNPs-3 exhibited an average size of 246, 300, and 328 nm, respectively. Additionally, they also formed with homogeneity particles as depicted from polydispersity index values (PDI). The PDI values of StNPs-1, StNPs-2, and StNPs-3 are 0.298, 0.177, and 0.262, respectively.

In vivo investigation of the potential properties of the different concentrations of StNPs loaded with NIQBD against MTX-induced inflammation in the lung and liver showed a statistically substantial increase in levels of reduced glutathione (GSH) accompanied by a significant decrease in levels of oxidants such as malondialdehyde (MDA), nitric oxide (NO), advanced oxidation protein product (AOPP), matrix metalloproteinase 9/Gelatinase B (MMP-9), and levels of inflammatory mediators including interleukin 1-beta (IL-1β), nuclear factor kappa-B (NF-κB) in both lung and liver tissues, and a significant decrease in levels of plasma homocysteine (Hcy) compared to the MTX-induced inflammation group. The highly significant results were obtained by treatment with a concentration of 200 mg/mL.

Histopathological examination supported these results, where treatment showed minimal inflammatory infiltration and congestion in lung tissue, a mildly congested central vein, and mild activation of Kupffer cells in liver tissues.

**Conclusion:**

Combining the treatment of MTX with natural antioxidant supplements may help reducing the associated oxidation and inflammation.

## Introduction

Methotrexate (MTX, 4-amino-4-deoxy-N10-methyl pteroylglutamic acid) an analog of aminopterin (4-amino-pteroylglutamic acid) is a strong cytotoxic drug that was first created for the treatment of cancer and is now utilized in non-neoplastic disorders as an anti-inflammatory agent and immunosuppressant. As one of the few disease-modifying antirheumatic medicines available, the most often used medication in the second line therapy of rheumatoid arthritis (RA) [1].

As a medication, MTX promotes the release of adenosine into the extracellular space at low dosages utilized for RA therapy [2]. Adenosine is a powerful anti-inflammatory autacoids that inhibits neutrophil adherence to endothelium and fibroblasts in response to MTX [2]. In addition, MTX suppresses malignant cell growth, largely by preventing the de novo synthesis of purines and pyrimidines [2].

MTX has possible adverse reactions and may generate toxicity in many organs despite its antineoplastic, anti-inflammatory, and immunosuppressive properties [3]. Renal toxicity, hepatotoxicity, and neurotoxicity have all been reported as side effects of MTX. Even at low doses, MTX disrupts the bone marrow, the mucous membrane of the gut,hepatic toxicity [4] and can occasionally induce pneumonitis [5, 6].

To reduce the adverse effects associated with present therapies and increase patient compliance, novel therapeutic techniques, such as the employing of all-natural compounds possessing anti-inflammatory qualities, must be developed [7–9].

In last decades, nature is fruitful and active area for many pharmaceutical compounds encouraging researchers to discover and develop compounds to reach the best and optimum activity against several diseases [10].

**Scheme 1:**
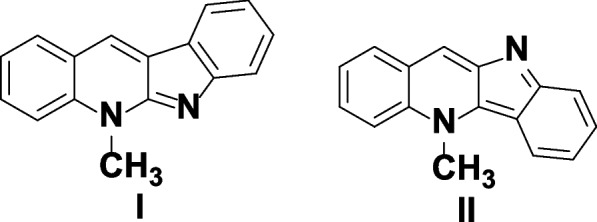
Structures of Neocrytolepine **I **and Cryptolepine **II**

A promising plant appeared to be *Cryptolepis sanguinolenta*, the roots of this climbing shrub are used in Central and West Africa in traditional medicine and it contains indoloquinoline alkaloids [11]. These tetracyclic alkaloids are formed by fusion of indole and quinoline with different sites and orientation. Neocryptolepine I (5-methyl-5H-indolo[2,3-b] quinoline) and its region isomer cryptolepine II (5-methyl-5H-indolo[3,2-b]quinoline) presented in (**Scheme** 1) have potent biological activity as anticancer, antimalarial and antimicrobial. Many structural modification and optimization were carried out to the lead core aiming to enhance and reach the best pharmaceutical properties for these alkaloids [10]. In addition, it was reported that upon in vitro inflammatory stimulation, cryptolepine has been demonstrated to reduce nitric oxide generation and nuclear factor-kappa B (NF-ĸB) DNA binding [12].

In order to improve drug effectiveness and address various problems with conventional medications, such as poor water solubility, limited bio-availability, and non-specific transport in the body, nanotechnology in drug delivery would be a promising strategy [13].

To enhance the solubility and bioavailability of neocryptolepine core, the preparation of hydrochloride salt of N1-(5-methyl-5H-indolo[2,3-b]quinolin-11-yl)benzene-1,4-diamine was performed in nanoscale was synthesized.

Combination between chemotherapeutic drugs and natural antioxidant products could potentially minimize these negative effects [14].

Based on this perspective, this research aimed for synthesizing N1-(5-methyl-5H-indolo[2,3-b]quinolin-11-yl)benzene-1,4-diamine hydrochloride (NIQBD), preparation of soluble starch nanoparticles (StNPs) loaded with different concentrations of NIQBD, and investigating the potential of StNPs that loaded with different concentrations of NIQBD in protecting lung and liver against MTX-induced inflammation.

## Materials and methods

### NMR analysis

^1^H NMR and ^13^C NMR analysis were used for the evaluation of the synthesized compound (NIQBD) 4 with 400 MHz and 100 MHz Bruker respectively. The FTIR spectrometer was carried out at Applied Nucleic acid research Center, Faculty of Science, Zagazig University using AlphaBruker ATR mode. The sample was prepared by using the KBr pellets technique. The Commercially available starting materials and solvents such as dimethyl formamide (DMF), and dimethyl sulfoxide (DMSO) were purchased from Sigma Aldrich and used as received without further purification. The key starting intermediate, 11-chloroneocryptolepine **1** was prepared according to our reported method [10]. Soluble starch was purchased from Doummar & Sons Co. Industrial City, Syria. Sodium hydroxide was purchased from El Nasr Pharmaceutical Chemicals, Egypt.

Methotrexate (50 mg) was purchased from MYLAN, USA. Homocystein standard (HPLC grade) was purchased from Sigma (St. Louis, USA). Other chemicals and reagents were of analytical grade (Sigma, St. Louis, USA).

The study was done on male Sprague–Dawley rats (140 ± 20 g). Rats were grouped and kept under controlled light and temperature, with unlimited access to standardized laboratory rat diet and water sources. Each group had eight rats. The experiments were carried out in accordance with the Ethics Committee of the National Research Centre's (NRC) guidelines for the care, use, and handling of laboratory animals, as well as the guidelines of the National Institutes of Health Guide for the Care and Use of Laboratory Animals.

### Synthesis of N1-(5-methyl-5H-indolo[2,3-b]quinolin-11-yl)benzene-1,4-diamine hydrochloride 4 (NIQBD)

Step1: To equimolar ratio of 11-chloroneocryptolpine **1** (3.75 g, 1 mmol) and 1.4-phenylene diamine **2** (1.52 g, 1 mmol) were added (2 mL) of DMF and triethyl amine (0.3 mL, 3 mmol) at room temperature. The above mixture was refluxed for 4 h till the complete consumption of the reactants as monitored by thin layer chromatography (TLC). After cooling down the reaction mixture to R.T., it was poured into ice water to afford yellow solid precipitate. The precipitate was filtered off, washed with water and dried to yield the free amine **3**.

Step2: The free amine 3 (0.58 g, 87%) was dissolved in methanol (2 mL) and cooled. The cold methanol solution was acidified by slowly adding 1 M of HCl until the formation of the hydrochloride salt **4**. The precipitated salt was filtered off dried to afford **4** in pure form.

Yellowish brown solid, yield (0.5 g,75%), FT-IR (KBr) cm^−1^ υ: 3359 (NH+NH_2_, overlapped),3044 (CH_Ar_), 2897 (CH), 1616(C = C), 1594 (C = N).^1^H NMR (DMSO d_6_-400 MHz) δ: 4.39 (s, 3H, N-CH3), 6.84–8.27 (m, 12H, CH_Ar_), 10.87 (s, 1H, NH_2_),13.25 (s, 1H, NH). ^13^C NMR (DMSO, d_6_-100 MHz) δ: 36.27, 105.63, 111.70, 112.80, 121.66, 122.10, 123.23, 126.99, 127.25, 138.37, 140.94, 146.08, 148.91.

### Preparation of soluble starch nanoparticles loaded with different concentrations of NIQBD

Initially, different concentrations of NIQBD (50 mg, 100 mg, and 200 mg) were vigorously dispersed into 5 mL of DMSO for 15 min at ambient temperature using ultrasonication probe. Secondly, soluble starch (St, 0.2 g) was gelatinized at 80 °C in deionized water (50 mL) containing 0.02 g of NaOH solution. Then, the dispersed NIQBD was added drop by drop under constant stirring for 5 min at 60 °C, after gelatinization the samples were sonicated for 30 min at room temperature. Thirdly, NIQBD loaded StNPs was separated using absolute ethanol followed by centrifugation process. The precipitated NIQBD loaded StNPs placed in refrigerator at -80 °C and dried using freeze drying at -80 °C for 24 h. Finally, the resultant samples of StNPs loaded with NIQBD were ground to fine powder and kept for further analysis and application. For application, 0.1 g of the resultant powder was dispersed in 10 mL of deionized water and kept under sonication for another 15 min before application. The three obtained StNPs loaded with 50 mg, 100 mg and 200 mg of NIQBD were nominated as StNPs-1, StNPs-2 and StNPs-3, respectively. For comparison, StNPs without NIQBD loading was prepared and coded as StNPs.

#### Characterization of StNPs loaded with different concentrations of NIQBD

The examination of particle for the prepared nanoparticles shape (stNPs-1, StNPs-2 and StNPs-3) was carried out using Transmission electron microscopy (TEM, JEOL, Japan). Meanwhile, Zetasizer (Malvern Instruments, UK) was used to investigate the particle size and polydispersity index (PDI) of StNPs-1, StNPs-2 and StNPs-3. The concentration of NIQBD in the supernatant was evaluated to determine the encapsulation efficiency of NIQBD loaded StNPs. The suspension was centrifuged at 12,000 rpm for 30 min after NIQBD loading, and the supernatant was measured at 318 nm with a UV–vis spectrophotometer. Different concentrations (2–10 μg/mL) were utilized to plot the standard calibration curve for NIQBD. The encapsulation efficiency (EE%) was calculated using the mathematical formulas below (Eq. 1).

1 $$\mathbf E\mathbf n\mathbf c\mathbf a\mathbf p\mathbf s\mathbf u\mathbf l\mathbf a\mathbf t\mathbf i\mathbf o\mathbf n\boldsymbol\;\mathbf e\mathbf f\mathbf f\mathbf i\mathbf c\mathbf i\mathbf e\mathbf n\mathbf c\mathbf y(\mathbf E\mathbf E\%)=\mathbf T\mathbf a-\mathbf T\mathbf b/\mathbf T\mathbf a\boldsymbol\;\mathbf x\boldsymbol\;100$$

In which, Ta refers to the total amount of NIQBD utilized for encapsulation. Meanwhile, Tb refers to the concentration of NIQBD in the supernatant.

### *Determination of the median lethal dose (LD*_*50*_*) of NIQBD loaded StNPs*

Twelve male Sprague–Dawley rats were used. At the first stage, nine animals were divided into three groups of three animals each. Each group of animals was administered different doses (100, 300, and 600 mg/kg) of NIQBD loaded StNPs. The animals were observed for 24 h to monitor their behavior as well as determine if mortality would occur. At the second stage, male Sprague–Dawley rats distributed into three groups of one animal each. Then the LD_50_ is calculated by the formula (Eq. 2) [15].

According to the LD_50_ assessment, the highest dose that gave no mortality was 300 mg/kg and the lowest dose that produced mortality was 600 mg/kg. The median lethal dose (LD_50_) of NIQBD loaded StNPs was 424.26 mg/kg body weight.

2 $${\mathbf{L}\mathbf{D}}_{50}=\sqrt{\left({\mathbf{D}}_{0}\times {\mathbf{D}}_{100}\right)}$$

Where: D0 = Highest dose that gave no mortality, D100 = Lowest dose that produced mortality.

### Induction of inflammation by Methotrexate

Rats were injected intraperitoneally with Methotrexate (MTX) in a dose of 14 mg/kg, as a single dose/week for 2 consecutive weeks according to Coleshowers et al. [16].

### Experimental design

48 male Sprague–Dawley rats were grouped into six groups (8 rats for each group) as follow: Control group: healthy rats received vehicle. Inflammatory group: Rats received MTX (14 mg/kg) intraperitoneally as a single dose/week for 2 consecutive weeks [16]. Treated group I: Rats received MTX (14 mg/kg) intraperitoneally as a single dose/week for 2 consecutive weeks [16], rats co-administrated 0.5 mL of StNPs daily using oral gavage orally for four weeks. Treated group II: Rats received MTX (14 mg/kg) intraperitoneally as a single dose/week for 2 consecutive weeks [16], rats co-administrated 0.5 mL of StNPs-1 (StNPs encapsulated NIQBD in a concentration of 50 mg/mL) daily using oral gavage orally for four weeks. Treated group III: Rats received MTX (14 mg/kg) intraperitoneally as a single dose/week for 2 consecutive weeks [16], rats co-administrated 0.5 mL of StNPs-2 (StNPs encapsulated NIQBD in a concentration of 100 mg/mL) daily using oral gavage orally for four weeks. Treated group IV: Rats received MTX (14 mg/kg) intraperitoneally as a single dose/week for 2 consecutive weeks [16], rats co-administrated 0.5 mL of StNPs-3 (StNPs encapsulated NIQBD in a concentration of 200 mg/mL) daily using oral gavage orally for four weeks.

After four weeks, rats were kept fasting for 12 h then blood samples were collected from the retro-orbital venous plexus to assess the level of homocystein. Rats were euthanized by decapitation; Lung and liver specimens were prepared for tissue homogenates for evaluation of biochemical analysis, histopathological, and immunohistochemical examination.

### Tissue preparation

Lung and liver tissues were excised immediately, washed with ice-cold saline, and blotted dry. Portions of the left lung and liver from all groups were homogenized in phosphate-buffered saline (PBS) at a 10% (w/v) dilution, centrifuged at 4000 rpm for 15 min at 4 °C, and the supernatants were frozen at -80 °C for further biochemical analysis. Histopathologic examinations were performed on the other portion of lung and liver tissues.

### Biochemical analysis

#### Determination of oxidants and anti-oxidants

Malondialdehyde (MDA), nitric oxide (NO), and reduced glutathione (GSH) were evaluated colorimetrically in lung and liver tissue homogenate as described by Ruiz-Larrea et al. [17], Moshage et al. [18], and Ellman et al. [19] respectively. Advanced oxidation protein product (AOPP) was evaluated in lung and liver tissue homogenate using enzyme-linked immunosorbent assay (ELISA). The kit was purchased from (SUNLONG, China). Procedures were followed according to the manufacturer’s instructions.

### Determination of inflammatory markers

Matrix metalloproteinase 9/Gelatinase B (MMP-9), interleukin 1-beta (IL-1β), and nuclear factor kappa-B(NF-κB) were evaluated in lung and liver tissue homogenate using enzyme-linked immunosorbent assay (ELISA). The kit was purchased from (SUNLONG, China). Procedures were followed according to the manufacturer’s instructions.

### Determination of plasma homocysteine by HPLC

HPLC was used for the assessment of plasma homocysteine (Hcy) [20, 21].

### Sample extraction

Four hundred microliters (400 μL) of the sample were mixed with 30 μL of 1.2 M trichloroacetic acid (TCA). The mixture was then incubated on ice for 30 min to precipitate protein. Following incubation, the mixture was centrifuged at 3200 rpm and 4 °C for 20 min. The supernatants were subsequently filtered through a hydrophilic 0.45-μm polyvinylidene fluoride (PVDF) membrane filter.

### HPLC condition

Fifty microliters (50 μL) of the filtered supernatant was injected into the high-performance liquid chromatography (HPLC) system. Separation was achieved on a reversed-phase (RP) column with dimensions of 25 cm × 0.46 cm × 5 μm, packed with C18 stationary phase. The mobile phase consisted of sodium phosphate monobasic monohydrate (40 mmol/L), heptanesulfonic acid (8 mmol/L), and 18% (v/v) methanol. The pH was adjusted to 3 using phosphoric acid. The mobile phase was then filtered through a 0.45 μm membrane filter and eluted at a flow rate of 1 mL/min at 40 °C. A UV detector set at 260 nm was used for detection. The sample concentration was calculated from the standard curve.

### Histopathological examination

Animals were euthanized by decapitation. Livers and lungs were then excised, fixed in 10% formalin, dehydrated through a graded series of ethanol solutions, cleared in xylene, embedded in paraffin wax, and sectioned at 5 μm using a rotary microtome. The paraffin sections were stained with hematoxylin and eosin. Histopathological evaluation was performed using light microscopy, and photomicrographs were captured.

### Statistical analysis

Data are presented as mean ± standard error (SE). Statistical analysis was performed using SPSS software (version 16). Differences were considered statistically significant at p < 0.05.

## Results

### Synthesis of N1-(5-methyl-5H-indolo[2,3-b]quinolin-11-yl)benzene-1,4-diamine hydrochloride 4 (NIQBD)

**Scheme 2:**
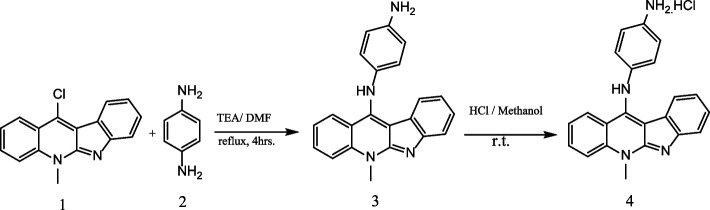
Synthesis of hydrochloride salt of **4**

To form the free amine of neocryptolepine derivative **3**, The as prepared 11-chloroneocryptoleine **1**, reacted with 1.4 phenylene diamine **2 **in equimolar ratio with presence of dimethylformamide (DMF) as a solvent and three times excess of triethyl amine (TEA) as a base under reflux to afford the free amine **3**in 87% yield. The free base **3 **was dissolved in 2 mL of methanol and cooled in ice bath and acidified by addition of few drops of 1 M hydrochloric acid (HCl) till the complete precipitation of the hydrochloride salt. The salt was filtered off, washed with methanol and dried to afford pure 4 as depicted in **Scheme** 2 [22].

The establishment of was elucidated by Fourier-transform infrared spectroscopy (FTIR) and nuclear magnetic resonance spectroscopy (NMR). The FTIR spectra showed υ_(NH)_ at 3359 cm^−1^ whereas υ_(CHAr)_ at 3044 cm^−1^ and υ_(C=C)_ at1616 cm^−1^. Furthermore,^1^H NMR the (*N*-CH_3_) group of neocryptolepine core were reported at δ: 4.39 ppm while in ^13^C NMR the (*N*-CH_3_) group showed at δ: 36.27 ppm.

### Characterization of NIQBD loaded soluble starch nanoparticles

The aim of our current work was designed to enhance the biomedical efficacy of NIQBD loaded StNPs. Via reacting soluble starch with sodium hydroxide (NaOH) at 80 °C, gelatinization of soluble starch was obtained. The use of absolute ethanol as a precipitating agent allowed NIQBD to be grafted into the gelatinized starch. The grafting of StNPs with NIQBD is triggered by ultrasonication waves. The encapsulation efficiency of NIQBD was found to be 93, 89,2 and 86,4%, respectively, for the prepared samples StNPs-1, StNPs-2, and StNPs-3, implying that NIQBD was encapsulated within StNPs.

**Fig. 1:**
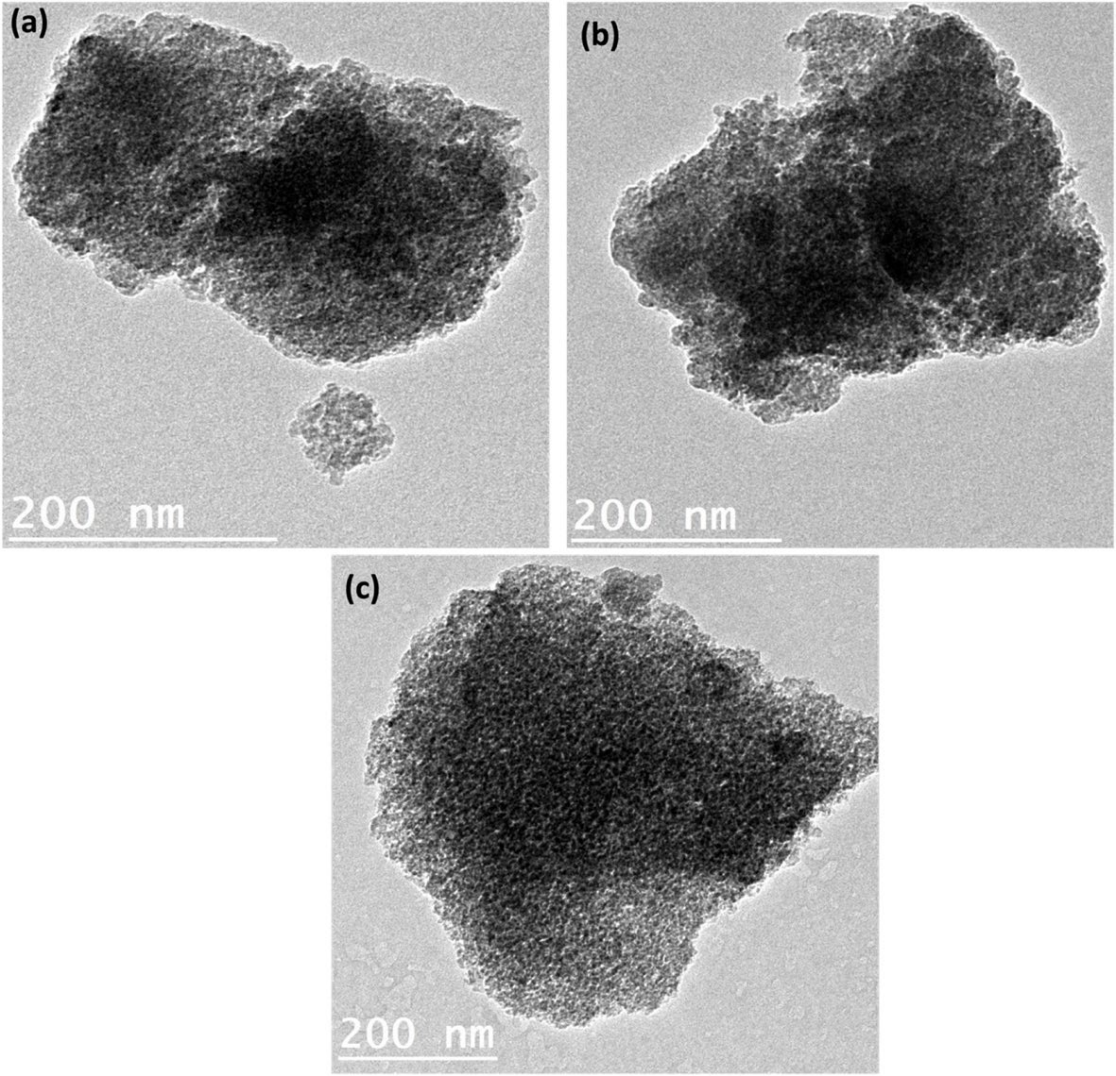
Particle shape of NIQBD loaded StNPs using TEM where: **a** StNPs-1, **b** StNPs 2 and **c** StNPs-3

The particle shape and homogeneity of NIQBD loaded StNPs were illustrated using TEM for the prepared samples; StNPs-1, StNPs-2 and StNPs-3 as can be seen in Fig. 1a, b, and c. All evaluated samples have nearly spherical shape. However, the particles tend to agglomerate together into clusters. As shown in Fig. 1, some NIQBD were deposited onto the surface of StNPs and tended to enlarge the particles of StNPs.

**Fig. 2:**
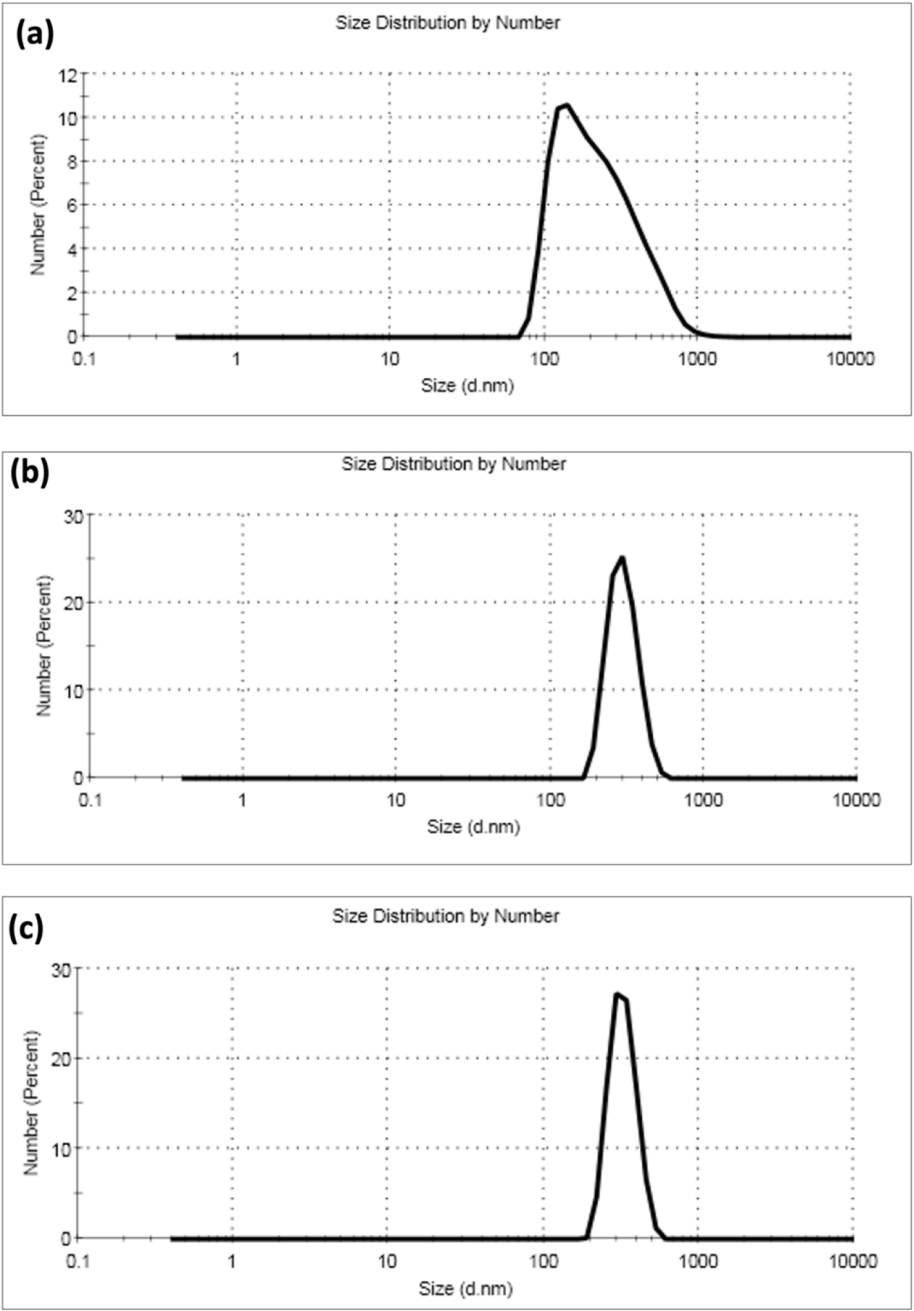
Average particle size of NIQBD loaded StNPs, **a** StNPs-1, **b** StNPs-2 and **c** StNPs-3

**Fig. 3:**
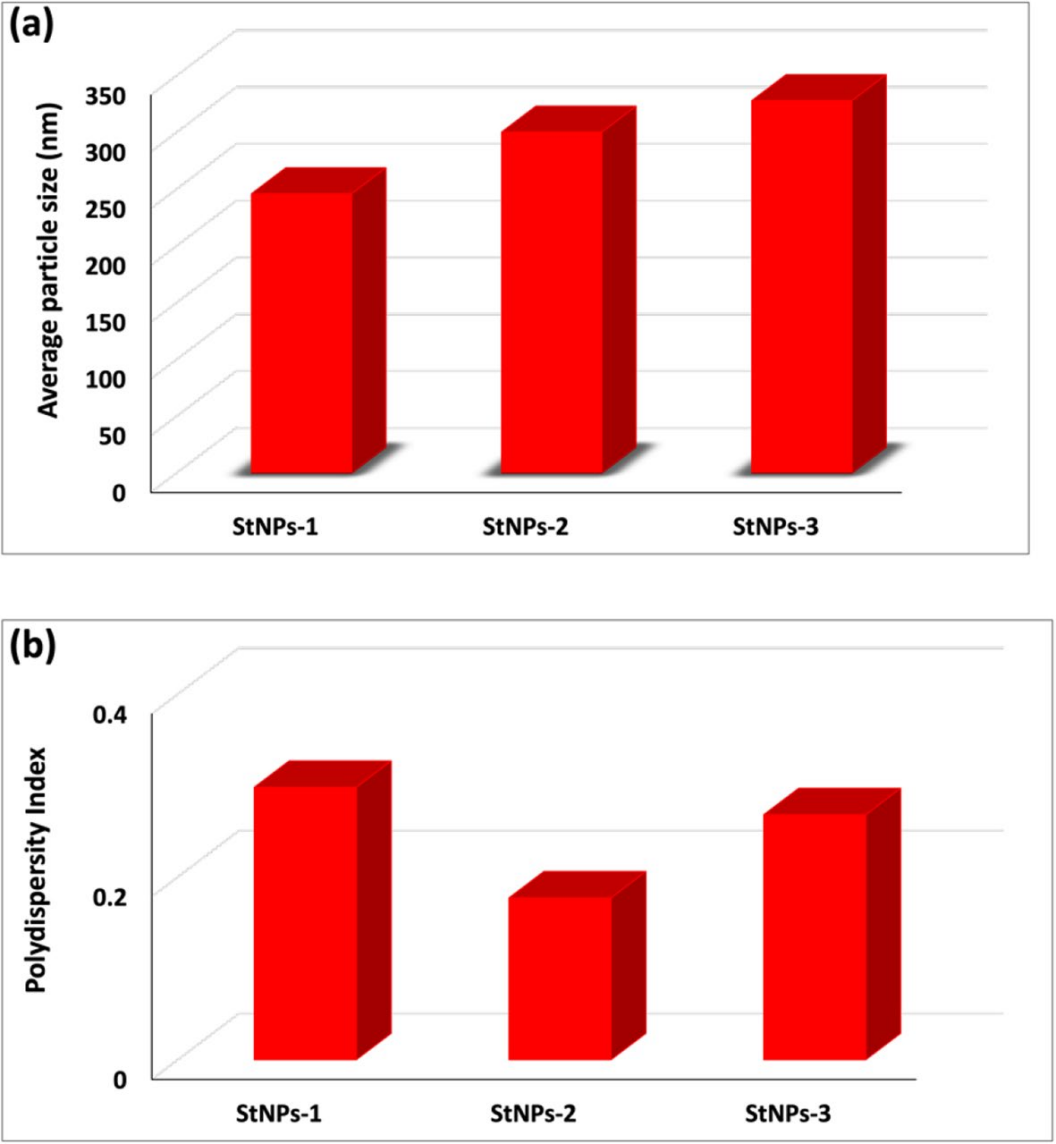
**a** Particle size and **b** PDI of NIQBD loaded StNPs

The particle size and polydispersity index (PDI) of the prepared NIQBD loaded StNPs ( StNPs-1, StNPs-2 and StNPs-3) were evaluated using dynamic light scattering (DLS). Figures 2 and 3 (**a**) illustrated the average size of NIQBD (different concentrations) loaded StNPs which recorded 246 nm for StNPs-1, 300 nm for StNPs-2, and 328 nm for StNPs-3.

Figure 3(b) displayed the polydispersity index (PDI) of the formed NIQBD loaded StNPs. As shown, all the prepared StNPs that loaded with different concentrations of NIQBD exhibited small PDI values. The PDI values of StNPs-1, StNPs-2 and StNPs-3 are 0.298, 0.177, and 0.262, respectively. All these PDI values are less than 0.5 which proved the homogeneity of the prepared NIQBD loaded StNPs.

### Biochemical analysis

#### Effect of MTX and different treatments on lung oxidants and anti-oxidants

Oxidative stress is a key indicator of cellular damage because it affects cell membrane permeabilization and impairs cellular functioning. To investigate the role of MTX as a reported oxidant, we estimated the levels of GSH, MDA, NO, and AOPP in lung tissues. Induction of MTX showed a marked decrease in the activity of lung GSH followed by a statistically substantial increase in the lung levels of MDA, NO, and AOPP in comparison to the control group (Table 1). In MTX group, the percent of change from the control group of GSH, MDA, NO, and AOPP were -55.4%, 525.8%, 288.7%, and 174.8% respectively as shown in Table 1.

**Table 1:** Lung oxidant and anti-oxidant levels in different studied groups

**Parameters**	GSH (mg / g tissue)	MDA (nmol /g tissue)	NO (μmol / g tissue)	AOPP (ng/g tissue)
**Groups**				
**Control**				
Mean ± SE	30.09 ± .87	39.8 ± 1.5	7.1 ± .37	13.5 ± .25
**MTX**				
Mean ± SE % of change from control group	13.41^a^ ± .15 -55.4%	249.1^a^ ± 1.7 525.8%	27.6^a^ ± .55 288.7%	37.1^a^ ± .58 174.8%
**MTX + StNPs**				
Mean ± SE % of change from MTX group	14.64^a^ ± .25 9.1%	213.2^a,b^ ± 1.4 -14.4%	21.5^a,b^ ± .58 -22.1%	25.7^a,b^ ± .25 -30.72%
**MTX + StNPs-1**				
Mean ± SE % of change from MTX group	17.19^a,b,c^ ± .46 28.1%	180^a,b,c^ ± 2.6 -27.7%	17.8^a,b,c^ ± .45 -35.5%	22.6^a,b,c^ ± .54 -39%
**MTX + StNPs-2** Mean ± SE				
% of change from MTX group	19.96^a,b,c^ ± .34 48.8%	119.9^a,b,c^ ± 2.2 -51.8%	15.3^a,b,c^ ± .53 -44.5%	20.5^a,b,c^ ± .46 -44.7%
**MTX + StNPs-3**				
Mean ± SE % of change from MTX group	23.44^a,b,c^ ± .38 74.7%	82.7^a,b,c^ ± 1.9 -66.8%	10.8^a,b,c^ ± .25 -60.8%	18^a,b,c^ ± .49 -51.4%

*P*^a^: significance compared to the control group, *P*^b^: significance compared to MTX group,* P*^*c*^: significance compared to MTX + StNPs group

Treatment with StNPs alone or StNPs loaded with NIQBD in different concentrations; StNPs-1, StNPs-2, or StNPs-3 showed a statistically substantial increase in the activity of lung GSH with a percent of change 9.1%, 28.1%, 48.8%, and 74.7% respectively from the MTX group (Table 1).

Compared to the MTX group, treatment with StNPs alone, StNPs-1, StNPs-2, or StNPs-3 showed a significant decrease in the levels of lung MDA with a percent of change -14.4%, -27.7%, -51.8%, -66.8% respectively from the MTX group (Table 1). In addition, levels of lung NO showed significant decrease with percent of change -22.1%, -35.5%, -44.5%, -60.8% respectively from the MTX group (Table 1). Levels of lung AOPP showed significant decrease compared to the MTX group upon the treatment with StNPs alone, StNPs-1, StNPs-2, or StNPs-3 with a percent of change -30.7%, -39%, -44.7%, -51.4% respectively from the MTX group (Table 1).

#### Effect of MTX and different treatments on lung inflammatory markers

In order to verify the mechanisms of inflammatory processes induced by MTX, we estimated the levels of MMP-9, IL-1β, and NF-kB in lung tissues. Our results showed that MTX induced inflammation resulted in a statistically substantial increase in the levels of lung MMP-9, IL-1β, and NF-kB compared to the control group. The percent of change of MMP-9, IL-1β, and NF-kB were 307%, 280%, and 66.7% respectively from the control group (Table 2).

**Table 2:** Lung inflammatory markers in different studied groups

**Parameters**	MMP-9 (ng/mg tissue)	IL-1β (pg/mg tissue)	NF-kB (pg/mg tissue)
**Groups**			
**Control**			
Mean ± SE	37.3 ± 1.2	15.5 ± .61	84.7 ± 1.5
**MTX**			
Mean ± SE % of change from control group	152^a^ ± 2.7 307%	58.9^a^ ± 1.3 280%	141.2^a^ ± .22 66.7%
**MTX + StNPs**			
Mean ± SE % of change from MTX group	143.6^a,b^ ± 1.4 -5.5%	52.1^a,b^ ± .51 -11.5%	138.6^a,b^ ± .43 -1.84%
**MTX + StNPs-1**			
Mean ± SE % of change from MTX group	135.2^a,b,c^ ± .67 -11%	45.7^a,b,c^ ± 1.0 -22.4%	134.9^a,b,c^ ± .33 -4.46%
**MTX + StNPs**			
Mean ± SE % of change from MTX group	129^a,b,c^ ± .96 -15%	33.5^a,b,c^ ± .92 -43.1%	129.5^a,b,c^ ± .46 -8.28%
**MTX + StNPs-3**			
Mean ± SE % of change from MTX group	78.9^a,b,c^ ± 1.3 -48%	26.3^a,b,c^ ± 1.1 -55.3%	115.6^a,b,c^ ± .51 -18.13%

*P*^a^: significance compared to the control group, *P*^b^: significance compared to MTX group,* P*^*c*^: significance compared to MTX + StNPs group

Treatment with StNPs alone, StNPs-1, StNPs-2, or StNPs-3 showed a significant decrease in the levels of lung MMP-9 with a percent of change -5.5%, -11%, -15%, -48% respectively from the MTX group (Table 2), levels of lung IL-1β showed significant decrease with percent of change -11.5%, -22.4%, -43.1%, -55.3% respectively from the MTX group (Table 2). Lung NF-kB showed significant decrease compared to the MTX group upon the treatment with StNPs alone, StNPs-1, StNPs-2, or StNPs-3 with a percent of change -1.84%, -4.46%, -8.28%, -18.13% respectively from the MTX group (Table 2).

#### Effect of MTX and different treatments on liver oxidants and anti-oxidants

The liver's pivotal role in drug metabolism and clearance renders it highly susceptible to the toxic effects of these pharmacological agents. The hepatotoxic manifestations of MTX have been attributed primarily to the induction of oxidative stress.

In comparison to the control group, MTX administration showed a significant decrease in the activity of GSH in the liver tissues accompanied with a statistically substantial increase in the levels of MDA, NO, and AOPP in liver tissues (Table 3). In MTX group, GSH, MDA, NO, and AOPP percent of change from the control group were -61.2%, 219%, 371.3%, and 199.3% respectively as shown in Table 3.

**Table 3:** Liver oxidant and anti-oxidant levels in different studied groups

**Parameters**	GSH (mg / g tissue)	MDA (nmol /g tissue)	NO (μmol / g tissue)	AOPP (ng/mg tissue)
**Groups**				
**Control**				
Mean ± SE	24.5 ± .37	270.6 ± 11.5	37.3 ± 1.5	15 ± .43
**MTX**				
Mean ± SE % of change from control group	9.5^a^ ± .18 -61.2%	864.7^a^ ± 6.3 219.5%	175.8^a^ ± 4.4 371.3%	44.9^a^ ± .85 199.3%
**MTX + StNPs**				
Mean ± SE % of change from MTX group	11.4^a,b^ ± .10 16.6%	749^a,b^ ± 5.8 -13.3%	127.7^a,b^ ± 1.2 -27.3%	33.8^a,b^ ± .63 -24.7%
**MTX + StNPs-1**				
Mean ± SE % of change from MTX group	13^a,b,c^ ± .35 36.8%	660.8^a,b,c^ ± 8.6 -23.5%	108^a,b,c^ ± 2.3 -38.5%	28.2^a,b,c^ ± .53 -37.1%
**MTX + StNPs-2**				
Mean ± SE % of change from MTX group	16.1^a,b,c^ ± .10 69.4%	548.3^a,b,c^ ± 6.0 -36.5%	72.2^a,b,c^ ± .71 -58.9%	24^a,b,c^ ± .30 -46.5%
**MTX + StNPs-3**				
Mean ± SE % of change from MTX group	20.5^a,b,c^ ± .59 115.7%	404^a,b,c^ ± 7.0 -53.2%	64.7^a,b,c^ ± 1.3 -63.1%	19.4^a,b,c^ ± .36 -56.7%

*P*^a^: significance compared to the control group, *P*^b^: significance compared to MTX group,* P*^*c*^: significance compared to MTX + StNPs group

Using StNPs alone, StNPs-1, StNPs-2, or StNPs-3 as a treatment showed a statistically substantial increase in the activity of liver GSH with a percent of change from the MTX group were 16.6%, 36.8%, 69.4%, and 115.7% respectively (Table 3).

Compared to the MTX group, treatment with StNPs alone, StNPs-1, StNPs-2, or StNPs-3 showed a significant decrease in the levels of liver MDA with a percent of change of the treated groups from the MTX group were -13.3%, -23.5%, -36.5%, -53.2% respectively (Table 3). In addition, levels of liver NO showed significant decrease with percent of change -27.3%, -38.5%, -58.9%, -63.1% respectively from the MTX group (Table 3).

Levels of liver AOPP showed significant decrease compared to the MTX group upon the treatment with StNPs alone, StNPs-1, StNPs-2, or StNPs-3 with a percent of change -24.7%, -37.1%, -46.5%, -56.7% respectively from the MTX group (Table 3).

### Effect of MTX and different treatments on liver inflammatory markers

Obtained results revealed that MTX-induced inflammation showed a statistically substantial increase in the levels of liver MMP-9, IL-1β, and NF-kB compared to the control group. The percent of change of MMP-9, IL-1β, and NF-kB were 169%, 155.8%, and 157.4% respectively from the control group (Table 4).

**Table 4:** Liver inflammatory markers in different studied groups

**Parameters**	MMP-9 (ng/mg tissue)	IL-1β (pg/mg tissue)	NF-kB (pg/mg tissue)
**Groups**			
**Control**			
Mean ± SE	38.5 ± .91	17.2 ± .54	99.2 ± 4.1
**MTX**			
Mean ± SE % of change from control group	103.6^a^ ± 2.1 169%	44^a^ ± .82 155.8%	255.4^a^ ± 1.8 157.4%
**MTX + StNPs**			
Mean ± SE % of change from MTX group	92.7^a,b^ ± 1.1 -10.5%	36.8^a,b^ ± .76 -16.3%	241.9^a,b^ ± 1.1 -5.2%
**MTX + StNPs-1**			
Mean ± SE % of change from MTX group	81.9^a,b,c^ ± 1.2 -20.9%	28.8^a,b,c^ ± .80 -34.5%	234.5^a,b,c^ ± 1.3 -8.1%
**MTX + StNPs-2**			
Mean ± SE % of change from MTX group	68^a,b,c^ ± 1.5 -34.3%	24.4^a,b,c^ ± .48 -44.5%	213.6^a,b,c^ ± 1.7 -16.3%
**MTX + StNPs-3**			
Mean ± SE % of change from MTX group	52.2^a,b,c^ ± 1.0 -49.6%	21^a,b,c^ ± .40 -52.2%	168^a,b,c^ ± 2.2 -34.2%

*P*^a^: significance compared to the control group, *P*^b^: significance compared to MTX group,* P*^*c*^: significance compared to MTX + StNPs group

Treatment with StNPs alone, StNPs-1, StNPs-2, or StNPs-3 showed a significant decrease in the levels of liver MMP-9 with a percent of change from the MTX group were -10.5%, -20.9%, -34.3%, -49.6% respectively (Table 4), levels of liver IL-1β showed significant decrease with percent of change from the MTX group were -16.3%, -34.5%, -44.5%, -52.2% respectively (Table 4).

Levels of NF-kB in liver tissues showed significant decrease compared to the MTX group upon the treatment with StNPs alone, StNPs-1, StNPs-2, or StNPs-3 with a percent of change –5.2%, -8.1%, -16.3%, -34.2% respectively from the MTX group (Table 4).

#### Effect of MTX and different treatments on serum Homocysteine

Serum homocystein levels showed a highly statistically substantial increase in MTX-induced inflammation group compared to the control group with a percent of change 1690% from the control group (Table 5).

**Table 5:** Serum homocystein levels in different studied groups

**Parameters**	Hcy (ng/ml)
**Groups**	
**Control**	
Mean ± SE	3.1 ± .10
**MTX**	
Mean ± SE % of change from control group	55.5^a^ ± 1.5 1690%
**MTX + StNPs**	
Mean ± SE % of change from MTX grou	43^a,b^ ± 1.1 -22.5%
**MTX + StNPs-1**	
Mean ± SE % of change from MTX group	31.5^a,b,c^ ± 1.0 -43.2%
**MTX + StNPs-2**	
Mean ± SE % of change from MTX group	24.6^a,b,c^ ± 1.1 -55.6%
**MTX + StNPs-3**	
Mean ± SE % of change from MTX group	11.5^a,b,c^ ± .76 -79.2%

*P*^a^: significance compared to the control group, *P*^b^: significance compared to MTX group,* P*^*c*\^: significance compared to MTX + StNPs group

On the other hand, levels of serum homocystein showed a significant decrease by using StNPs alone, StNPs-1, StNPs-2, or StNPs-3 as treatments, with percent of change from the MTX group -22.5%, -43.2%, -55.6%, and -79.2% respectively (Table 5).

### Histopathological results

#### Effect of MTX and different treatments on lung tissue

**Fig. 4:**
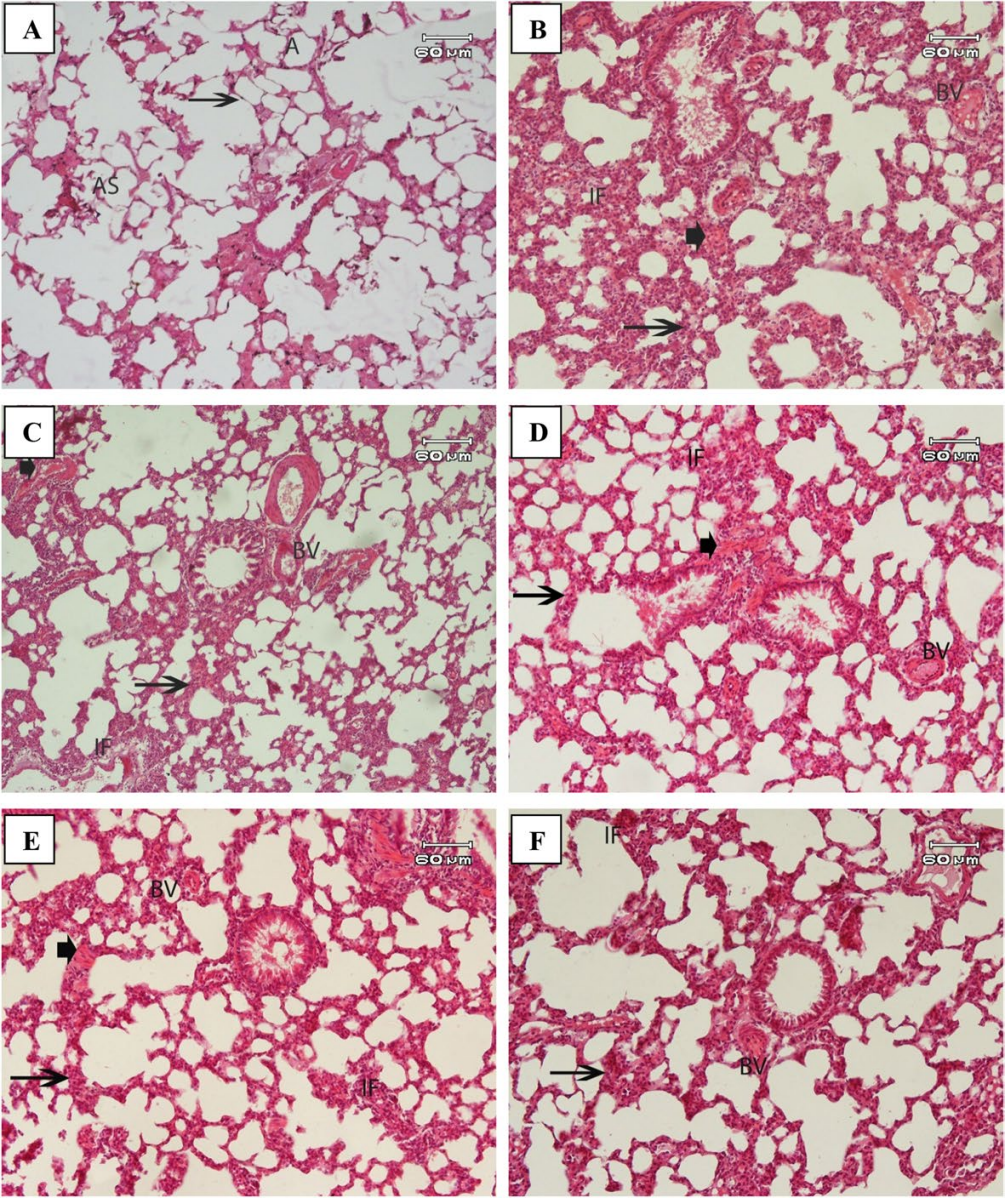
A micrograph of rat lung of: **A** control group showing normal architecture including alveolar sacs (AS), numerous normal regular alveoli (A) thin interalveolar septa (arrows). **B** MTX induced inflammation group showing disruption of normal lung architecture and distortion of the interalveolar septa with thickening (arrow), the pulmonary blood vessel showed dilatation and congestion (BV). Inflammatory cellular infiltration in perivascular (IF), and interstitial tissue with haemorrhage into alveolar spaces (arrowhead) were found. **C** MTX induced inflammation treated with StNPs showing moderated improvement with reduction of thickening of the interalveolar septa (arrow) and inflammatory cell infiltration (IF) and interstitial tissue with haemorrhage into alveolar spaces (arrowhead), blood vessel dilatation and congestion (BV). **D** MTX induced inflammation treated with StNPs-1group (low concentration) showing reduce thickening of the interalveolar septa (arrow) with minimal inflammatory infiltration (arrowhead), interstitial tissue with haemorrhage into alveolar spaces (arrowhead) and dilatation and congestion (BV). **E** MTX induced inflammation treated with StNPs-2 group (medium concentration) showing reduced thickening of the interalveolar septa (arrow) with minimal inflammatory infiltration (IF) and interstitial tissue with haemorrhage into alveolar spaces(arrowhead) and blood vessel dilatation and congestion (BV). **F** MTX induced inflammation treated with StNPs -3group (high concentration) showing marked reduced thickening of the interalveolar septa (arrow) with minimal inflammatory infiltration (IF) and blood vessel dilatation and congestion (BV). H&E stain, Scale bar: 60 μm

Microscopic examination of lung sections from control rats revealed normal lung architecture with thin interalveolar septa, clear rounded or polygonal alveoli, and alveolar sacs. The interalveolar spaces were extremely narrow, and the interstitial tissue contained blood vessels (Fig. 4A.

Histopathological evaluation of lung sections from MTX-treated rats showed disruption of the normal architecture. This disruption was characterized by apparent alveolar damage, including collapse and distortion of the interalveolar septa with thickening. Additionally, the pulmonary blood vessels exhibited dilatation and congestion, accompanied by extravasation of red blood cells into the alveolar lumen. Inflammatory cell infiltration was observed in the perivascular and interstitial tissues, with hemorrhage into the alveolar spaces (Fig. 4B).

In the group received MTX and treated with StNPs revealed moderated improvement with reduction of thickening of the interalveolar septa and inflammatory cell infiltration and congestion of interstitial vessels (Fig. 4C).

However, MTX and treated with StNPs-1 revealed ameliorated by administration of different doses of StNPs-1, StNPs-2, and StNPs-3 evidenced by a reduction in interalveolar septal thickening, a histological hallmark of improvement with minimal inflammatory infiltration and congestion of interstitial vessels in dose dependent manner however the group of StNPs-3 (high concentration) is better than StNPs-1 and StNPs-2 (low & medium concentrations) (Fig. 4D, E and F respectively).

#### Effect of MTX and different treatments on liver tissue

**Fig. 5:**
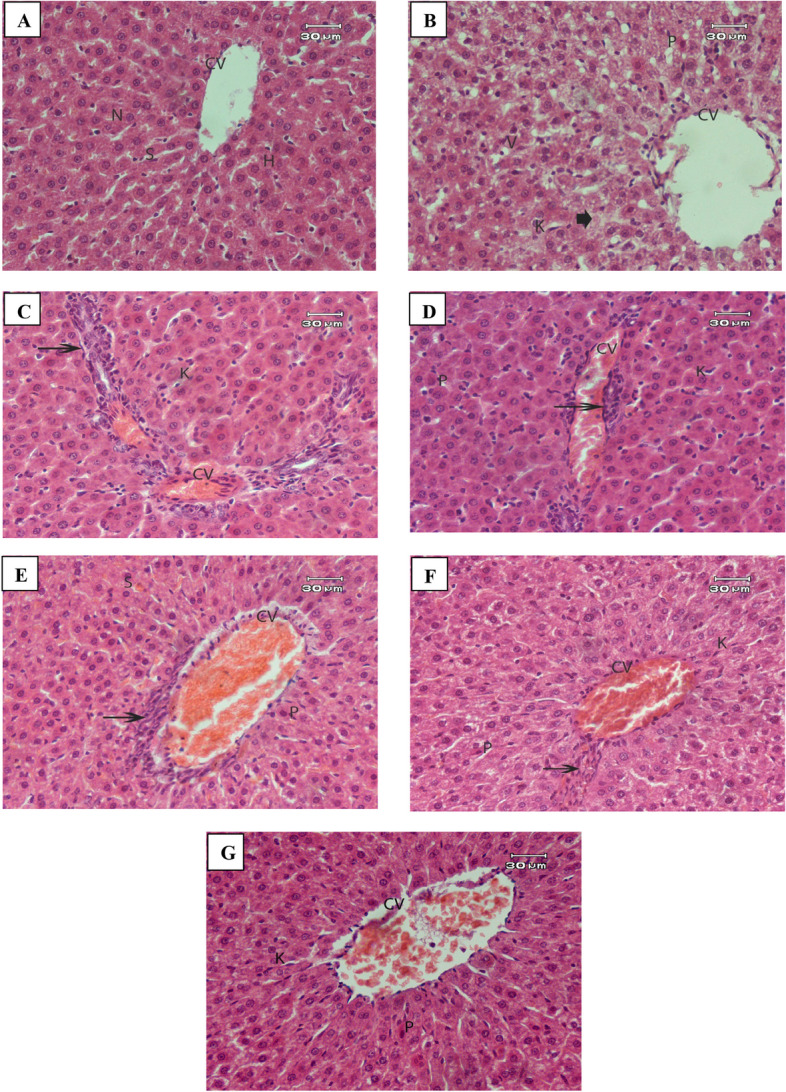
A micrograph of rat liver of: **A** control group showing hepatic architecture. Central vein (CV), hepatocytes (H), blood sinusoids (S) and nucleus (S) are shown. **B & C** MTX induced inflammation model group showing **B** degeneration changes with eosinophilic cytoplasm, necrotic areas (arrowhead), deeply stained pyknotic nuclei (P) with mild activation of Kupffer cells (K). **C** Showing congested central vein (CV) associated with moderate inflammatory cell infiltration (arrow). Deeply stained pyknotic nuclei (P) and mild activation of Kupffer cells (K) were seen. **E** MTX induced inflammation treated with StNPs group showing moderate improvement. Mild inflammatory cell infiltration (arrow) around the congested central vein (CV), mild activation of Kupffer cells (K) and pyknotic nuclei were noticed (P). **F** MTX induced inflammation treated with StNPs-1 (low concentration) group showing moderate improvement with inflammatory cell infiltration (arrow) around congested central vein (CV), mild haemorrhage in between blood sinusoids (S) and pyknotic nuclei (P). **G** MTX induced inflammation treated with StNPs-2 (medium concentration) group showing an improvement associated with small area of inflammatory cell infiltration (arrow) around congested central vein (CV). Mild activation of Kupffer cells (K) and pyknotic nuclei (P) were seen. **H** MTX induced inflammation treated with StNPs-3 (high concentration) group showing an improvement associated with mild congested central vein (CV), mild activation of Kupffer cells (K) and pyknotic nuclei (P)**.** H&E stain, Scale bar: 30 μm

Microscopic examination of liver sections from the control group revealed normal morphology with regular arrangement of hepatocytes, distinct nuclei, and patent sinusoids (Fig. 5A.

In the group that received MTX, hepatocytes exhibited vacuolation, disruption of the normal hepatic architecture, and increased evidence of necrosis (Fig. 5B). On the other hand, moderate accumulation of inflammatory cells around the congested central veins, most sinusoids showed numerous Kupffer cells and pyknotic nuclei (Fig. 5C).

Liver of the MTX group that treated with StNPs showed moderated improvement. Mild inflammatory cells infiltration around the congested central veins, mild activation of Kupffer cells and pyknotic nuclei were noticed (Fig. 5D).

The liver of MTX group that treated with StNPs-1 showed a moderated improvement of tissue. Mild inflammation around the congested central veins, mild haemorrhage in between blood sinusoids and pyknotic nuclei were seen (Fig. 5E).

MTX group that treated with StNPs-2 reversed the morphological changes caused by inflammation agent resulting in the restoration of normal liver architecture, reduced inflammatory cell infiltration, and preserve distinct lobular structure (Fig. 5F).

MTX group administration StNPs-3 attenuated congestion of central vein in liver tissues, reduce the number of the infiltration of inflammatory cells with hepatocyte arrangement closely resembled that of a normal liver (Fig. 5G).

## Discussion

Methotrexate (MTX) is a versatile drug with applications in both oncology and rheumatology. It is widely used to treat various cancers, including leukemia, breast cancer, head and neck cancers, and carcinomas, while also being effective in managing rheumatoid arthritis (RA) at lower doses. During the therapeutic stages, serious side effects have been documented in patients including acute or sub acute respiratory failure [23–26] and hepatotoxicity [4].

Our findings showed that the induction of MTX resulted in a statistically substantial increase in the levels of oxidants; MDA, NO, and AOPP accompanied with a significant decrease in the levels of anti-oxidants (GSH) in the tissues of both lung and liver. In accordance with our findings, research suggests that MTX treatment can cause a decrease in antioxidant enzyme levels and a subsequent rise in free radicals, as evidenced by various reports [27, 28].

The current investigation reported that the administration of MTX significantly decrease GSH concentrations, the antioxidant enzyme, in the tissues of the lung and liver compared to the normal group. MTX administration has been shown to inhibit cytosolic nicotinamide adenosine diphosphate (NADP)-dependent dehydrogenases along with various cellular anti-oxidants. NADPH is essential for maintaining cellular glutathione in a reduced state and prevents free radical releasing, that participates in the destruction of lipids, resulting in MDA elevated levels [29].

Our result supported by the findings of Drishya et al. who reported that the damage in organs caused by MTX is via reducing three major antioxidant enzymes: superoxide dismutase (SOD), glutathione peroxidase (GPx), and catalase (CAT) [30]. It additionally suppresses Glutathione (GSH) concentrations in the reduced form and boosts oxidation process via increasing MDA (a marker of membrane lipid peroxidation) and Nitric oxide (NO) free radicals [30]. The increase in reactive oxygen species (ROS) following MTX therapy might be due to an increase in apoptosis of altered T cells [31].

Several investigations have shown that ROS production plays a role in the onset of MTX toxicity. As a result, it was discovered that an increase in reactive oxygen and nitrogen species (ROS/RNS) paired with a decrease in antioxidant defense enzymes enhances the development of liver damage [27]. In addition, MTX accumulation in tissues cause suppression of RNA and DNA synthesis in cells, resulting in cellular arrest [32], pulmonary fibrosis [33], and hepatic fibrosis through activation of stellate cells [34].

Another potential mechanism underlying the toxic effects of MTX, which may further explain our findings, involves the role of reactive oxygen species (ROS). Although ROS play a crucial role in various cellular signaling pathways, excessive or mislocalized ROS can damage cells. Mitochondria are the main source of cellular ROS, and growing evidence suggests their involvement in signaling pathways that regulate cell survival and apoptosis. MTX reproted to induced mitochondrial injury and cytochrome c release in rat liver hepatocytes [27].

Our study demonstrates that MTX treatment increases ROS formation in hepatocytes. This increase in ROS leads to mitochondrial dysfunction, characterized by mitochondrial membrane potential collapse, swelling, decreased ATP and GSH levels, and cytochrome c release. Furthermore, the increased mitochondrial ROS oxidizes sulfhydryl groups surrounding the mitochondrial permeability transition pore (mPTP). This oxidation alters the mPTP conformation from closed to open, facilitating cytochrome c release and further mitochondrial damage [27].

Our data and other studies supported the findings that the effect of the excessive releasing of ROS by MTX via the inhibition of the antioxidant defense mechanism and consequently involved in the development of lung and liver damage [27, 35].

Among the potential damaging effects of MTX is the activation of apoptotic pathway as a consequence of oxidation [36]. MTX caused tissue damage by boosting inflammatory mediators such as TNF-α, IL-1β, and IL-6, elucidating the involvement of these inflammatory mediators in MTX toxicity [30].

As reported by our finding, MTX induced inflammation in both lung and liver tissues by significantly increasing levels of MMP-9, IL-1β, and NF-ĸB compared to the control group. In the same line, it was reported that MTX-induced is associated to the raised production of inflammation-related cytokines such as TNF-α, IL-1, IL-6, and IL-18 [37]. The excessive production of ROS promotes inflammation by inducing NF- κB. The activation of upstream kinases causes the oxidation and subsequent dissociation of inhibitor kappa B-alpha (IκB-α) from NF-κB. NF-κB enters the nucleus and stimulates the production of inflammatory cytokines, including interleukin-6 (IL-6) [38]. These pro-inflammatory cytokines damage endothelial cells, increasing vascular permeability and producing edema [39]. Furthermore, ROS promotes lipid peroxidation and protein oxidation, resulting in oxidation-specific epitopes, which are thought to be the primary inflammatory mediators [40].

Obtained results showed a statistically substantial increase in MMP-9 levels in lung and liver tissues. MMPs have the ability to degrade nearly all ECM proteins. Simultaneously, MMPs participate in the synthesis of protein messengers that involve chemokines and cytokines, thereby influencing their release as well as their action [41, 42]. MMP-2 and MMP-9, for example, may activate involved pro-fibrotic TGβ1 via proteolytic cleavage of TGF-1-bound latency-associated peptide (LAP) [43, 44]. MMPs are believed to be crucial in the oversight of the regeneration and repair of lung after injuries because of their ECM degrading activities, and the dysregulation of MMP activation pathway may be part of the imbalance between ECM depositions versus degradation that characterizes fibrogenesis [45].

In addition, in this study MTX-induced inflammation results in a statistically substantial increase in Hcy levels which was in accordance with previous studies [46, 47].which may be explained by the fact that the metabolites of MTX subsequently suppress methylene-tetrahydrofolate reductase (MTHFR), an enzyme essential for the formation of methyl THF from methylene THF and the subsequent formation of methionine from homocysteine [48]. MTHFR inhibition by MTX metabolite results in elevated intracellular oxidants and concentrations of homocysteine [49, 50].

MTX-induced cytotoxicity appears to diminish levels of glutathione (GSH) in many tissues [51, 52]. Because glutathione synthesis is linked to the methionine cycle via the condensation of homocysteine and the cleavage of cystathionine into cysteine, that interacts to glutamate and produces γ -gcs. γ-gcs or the transsulfuration pathway generates approximately 50% of glutathione.

Cellular oxidative stress blocks the silent mating type information regulation-2 homolog (Sirt1) from initiating nuclear factor erythroid 2-related factor 2 (Nrf2), which binds DNA to transcribe antioxidant genes, specifically γ-gcs [53]. γ-gcs is in charge of glutathione synthesis, stimulation, and cellular glutathione equilibrium. MTX suppresses methionine generation, resulting in hyper-homocysteinemia, which causes cellular oxidation that reduces Sirt1/Nrf2 mediated glutathione production and induce tissue injury [54].

In accordance with the biochemical analysis, our histopathological examination showed inflammatory cellular infiltration in perivascular and in the interstitial tissue with haemorrhage into alveolar spaces. In addition, the hepatocytes showed vacuolation in some hepatocytes, loss of hepatic architecture and increased signs of necrosis, accumulation of inflammatory cells around the congested central veins, and appearance of many Kupffer cells and pyknotic nuclei.

Our findings could be explained according to Imokawa et al. who reported that MTX causes epithelial cell cytotoxicity and, as a result, myofibroblast growth in the fibrosis region. Furthermore, MTX treatment enhanced the number of apoptotic cells in primary alveolar epithelial cells. As a result, the primary alveolar epithelial cells are more vulnerable to cell damage than fibroblasts. Interstitial inflammation, fibrosis, giant cells, and tissue eosinophils increased intra-alveolar macrophages in patients with MTX pneumonitis [3]. Interstitial inflammation, fibrosis, diffuse alveolar injury, and collagen formation were all seen in MTX-induced model animals. Taking their findings and ours into account, our model is comparable to patients with MTX induced inflammation in lung and liver tissues.

Taking our biochemical and the histopathological results, we can briefly state the suggested mechanism which supports and clarifies our obtained data where MTX cause alterations in the levels of oxidants and inflammatory mediators leading to a fibrotic response with dysfunction of cells, which is mediated by inflammation and apoptosis. MTX might cause cell damage, resulting in a degradation of the structure of the barrier underlying membranes, which leads to the infiltration and proliferation of myofibroblasts accompanying collagen accumulation [55].

A promising strategy for attenuating the systemic side effects of some drugs is to target the inflamed areas affected by these drugs through improving therapeutic efficacy, as local drug delivery results in higher drug concentrations in affected tissues and mitigates histological inflammation [56].

Considerable studies have conducted innovative drug delivery approaches that more effectively target the inflamed organ and increase the drug's shielding against gastrointestinal conditions. The use of nanocarriers has several benefits, including overcoming the different biological barriers that restrict therapeutic utilization of targets and improving the oral bioavailability of hydrophobic compounds [56]. Furthermore, the efficacy of some natural compounds in the form of nanoparticles (NPs) has been demonstrated to be enhanced in various diseases such as cancer [21, 57–59], liver fibrosis [60], Alzheimer [61], acute kidney injury [14],gastric ulcer [62], postmenopausal complications [63], and wound healing [64].

From this perspective, we aimed to prepare soluble starch nanoparticles (StNPs) loaded with different concentrations of N1-(5-methyl-5H-indolo[2,3-b]quinolin-11-yl)benzene-1,4-diamine hydrochloride (NIQBD) and investigated its effects against inflammation induced by MTX.

Our results reveal that the obtained data of the particle shape and size is suitable for the medical domains. As a result, the loading of NIQBD into StNPs was achieved with a small size, making it easier to penetrate through cells and increasing NIQBD delivery to the cell surface. According to Kumari et al. smaller nanoparticles (less than 500 nm) can effortlessly pass through the cell membrane and extend the time they spend circulating in biological fluids [65].

Results of DLS analyses showed that a homogeneous dispersion of the prepared nanoparticles. Furthermore, all the obtained data from PDI is observed to be less than 0.5 for the three prepared samples of NIQBD loaded StNPs indicated that the prepared NIQBD loaded StNPs was successfully prepared with good dispersion and uniformity.

Based on these promising results, the biochemical analysis of the groups treated with StNPs alone, StNPs-1, StNPs-2, or StNPs-3 showed a significant amelioration in the balance between oxidants and anti-oxidants where the levels of GSH showed statistically substantial increase in different treated groups accompanied with a significant decrease in levels of MDA, NO, and AOPP in both lung and liver tissues. In addition, a significant mitigation in the inflammatory cascades was observed where levels of lung and liver MMP-9, IL-1β, NF-ĸB, and serum Hcy showed significant decrease in treated groups compared to the MTX group. These results were supported by the histopathological examinations of the lung and liver tissues. The most significant results were obtained from the highest concentration of NIQBD loaded soluble starch nanoparticles (StNPs-3).

The use of natural substances with antioxidant characteristics reduce MTX-induced toxicity [66]. As a result, substances with antioxidant characteristics may improve the efficacy of cancer treatment drugs while also reducing systemic toxicity caused by chemotherapeutics. Drugs and their reactive metabolites have been shown to have different effects on gene expression and cellular homeostasis in lung and hepatocytes [67].

Neocryptolepine, an intriguing natural quinoline indole alkaloid, has received a lot of interest in recent years. The biological activity of neocryptolepine and its derivatives are diverse [22]. As a result, indolequinoline alkaloids are regarded as a promising framework for drug development and have the potential to be turned into useful medications [8, 22]. Cryptolepine and its derivatives showed anti-inflammatory efficacy both in vivo and in vitro. Because of its anti-inflammatory characteristics, cryptolepine decreases NO generation and DNA binding to NF-ĸB in vitro [68]. Olumayokun et al., proposes that cryptolepine may control inflammation through decreasing the stimulation of NF-ĸB binding with DNA and, as a result, suppressing the transcription of pro-inflammatory proteins regulated by NF-ĸB [69].

Natural products are crucial in the drug development and chemical manufacturing. The polycyclic quinoline chemical neocryptolepine was obtained from Cryptolepis sanguinolent. Thus, in this study a neocryptolepine derivative were produced by altering the structure of neocryptolepine in order to enhance their anti-oxidant and anti-inflammatory properties.

Furthermore, nanoparticles can actively gather in the inflammatory regions, benefiting from their higher intestinal permeability [70], which might improve the utilization of nanoparticles by infiltrating cells while minimizing systemic side effects [13]. Furthermore, because of their tiny dimensions, their ability to localize into specified damaged tissues might prove advantageous for the management of different diseases, especially Crohn's disease [71].

Earlier studies synthesized StNPs capable of delivering proteins and macromolecules into airway epithelial cells [72]. StNPs consist of positively charged, interconnected maltodextrin molecules (α1-4 linked D-glucose polymers) surrounding an inner core of negatively charged phospholipid (dipalmitoyl phosphatidylglycerol). This porous structure, with its lipophilic core, allows StNPs to actively penetrate cells via endocytosis and efficiently transport hydrophobic compounds [73].

11-hydroxycryptolepine was discovered to inhibit xanthine oxidase and the formation of superoxide anions significantly. The inclusion of a hydroxyl group in 11-hydroxycryptolepine, as opposed to the other alkaloids, is thought to be significant for the inhibition of xanthine oxidase and the formation of superoxide radicals [74].

The plant's main alkaloid, cryptolepine, inhibits *in vitro* nitric oxide generation and NF-ĸB DNA binding in response to stimuli that induce inflammation [75].

## Conclusion

Inflammation is critical for maintaining homeostasis. Uncontrolled inflammation, on the other hand, can progress to chronic inflammation and cause a variety of chronic disorders such as hepatitis, arthritis, and neurological disease.

Due to its beneficial pharmacological properties, neocryptolepine may be employed as a molecule in integrated medicine in the near future, with significant potential for protecting against MTX side effects. Herein, we anticipated that NIQBD loaded soluble starch nanoparticles is a promising delivering mechanism to mitigate MTX-induced oxidation and inflammation.

## Data Availability

No datasets were generated or analysed during the current study.
